# Writer’s Cramp

**Published:** 1882-03

**Authors:** 


### Writer's Cramp.

Wernicke has lately found a peculiarity which, if present in all instances, sheds an entirely new light on this affection. In an ordinary case reported to the Berlin Physiological Society (*Arch. f. Phys.*, 1881, p. 197), he observed an isolated paralysis of the extensor pollicis longus muscle, which muscle, according to Duchenne, is not immediately concerned in the act of writing. However, it is of decided influence on the position of the thumb, and hence the author believes the paralysis to be an etiological factor in the disease.

